# Brain Structural and Perfusion Signature of Amyotrophic Lateral Sclerosis With Varying Levels of Cognitive Deficit

**DOI:** 10.3389/fneur.2018.00364

**Published:** 2018-05-24

**Authors:** Dongchao Shen, Bo Hou, Yinyan Xu, Bo Cui, Pan Peng, Xiaolu Li, Hongfei Tai, Kang Zhang, Shuangwu Liu, Hanhui Fu, Jing Gao, Mingsheng Liu, Feng Feng, Liying Cui

**Affiliations:** ^1^Department of Neurology, Peking Union Medical College Hospital, Chinese Academy of Medical Sciences, Peking Union Medical College, Beijing, China; ^2^Department of Radiology, Peking Union Medical College Hospital, Chinese Academy of Medical Sciences, Peking Union Medical College, Beijing, China; ^3^Neuroscience Center, Chinese Academy of Medical Sciences, Beijing, China

**Keywords:** amyotrophic lateral sclerosis, cognitive impairment, frontotemporal dementia, voxel-based morphometry, arterial spin labeling, cerebral blood flow

## Abstract

**Objective:**

To characterize the patterns of brain atrophy and perfusion as measured by arterial spin labeling (ASL)-MRI, in amyotrophic lateral sclerosis (ALS) patients with varying levels of cognitive deficit, including ALS with frontotemporal dementia (FTD).

**Methods:**

A total of 55 ALS patients and 20 healthy controls (HCs) were included, and all participants underwent neuropsychological assessments and MRI scans. According to their cognitive performance, ALS patients were further subclassified into ALS with normal cognition (ALS-Cn, *n* = 27), ALS with cognitive impairment (ALS-Ci, *n* = 17), and ALS-FTD (*n* = 11). Voxel-based comparisons of gray matter (GM) changes and cerebral blood flow (CBF) were conducted among the subgroups.

**Results:**

The whole-brain comparisons of GM changes and CBF among ALS-Ci, ALS-Cn, and HCs were not significantly different. However, the ALS-FTD patients demonstrated a similar pattern of GM loss and hypoperfusion with more significant alterations in the left frontal and temporal lobe compared with the HCs, ALS-Cn, and ALS-Ci patients. Decreased CBF was found in many of the same brain areas wherein structural alterations occurred, although isolated GM loss and hypoperfusion were also observed. In addition, for both GM and CBF abnormalities, a similar pattern of changes was found in the comparisons of ALS-FTD vs. ALS-Ci, ALS-FTD vs. ALS-Cn, and ALS-FTD vs. HCs, with the differences being most significant between ALS-FTD and HCs.

**Conclusion:**

The cognitive status of ALS patients is associated with different patterns of GM changes and cerebral perfusion. ASL-MRI might be a useful tool with which to investigate the pathological burden of ALS and to disclose the early signature of possible cognitive impairment.

## Introduction

Amyotrophic lateral sclerosis (ALS) is a progressive neurodegenerative disease involving both upper and lower motor neurons with no effective cure. In addition to motor impairment, studies have found extramotor abnormalities in ALS including cognitive deficit, autonomic disorders, and extrapyramidal signs (1). Specifically, patients with ALS could display a spectrum of cognitive and behavioral deficits ranging from mild deficits to severe frontotemporal dementia (FTD) (2). TDP-43 inclusions within the central nervous system and expansion mutations in the *C9orf72* gene can be detected in both diseases. Thus, the clinical, pathological, and genetic overlap between ALS and FTD is widely recognized. Advances in the understanding of the spectrum of frontotemporal dysfunction in ALS have mandated the revision of the Strong criteria, and the term “ALS frontotemporal spectrum disorders” has now been adopted to characterize the expanded syndrome (3). Overall, an estimated 35–40% of individuals with ALS may display fully developed FTD, and 30–60% exhibit more subtle cognitive alterations involving executive domains, social cognition, language, or memory, or present with neuropsychiatric symptoms (4–8). These deficits are manifestations of the spectrum of disorders resulting from frontotemporal dysfunction in patients with ALS (3).

Neuroimaging investigations continue to provide *in vivo* pathological insights into the involvement of frontotemporal and limbic regions in ALS, thus lending support to a neurostructural overlap with FTD. Several imaging techniques, including voxel-based morphometry (VBM) or surface-based morphometry (SBM), diffusion tensor imaging (DTI), single-photon emission computed tomography (SPECT), and ^18^F-2-fluoro-2-deoxy-d-glucose-PET (FDG-PET), have helped to identify brain changes related to cognitive impairment in patients with ALS (9). Extensive gray matter (GM) abnormalities in ALS, beyond the motor system, have been reported in most studies (10). In VBM and SBM analyses, ALS-FTD patients exhibited GM loss in motor and somatosensory areas as well as the adjacent frontal and temporal cortices, and ALS patients with cognitive impairment (ALS-Ci) demonstrated GM changes in regions largely overlapping with those found in ALS-FTD, but these changes were less widespread (11, 12). The degree of subcortical GM alterations is also closely associated with neuropsychological impairment, with increasing basal ganglia pathology across the ALS/FTD spectrum (13). Degeneration of white matter (WM) fibers in extramotor areas was detected in ALS patients both with and without cognitive impairment but was more severe in ALS-Ci patients (14, 15). FDG-PET studies have reported a gradient of brain metabolic changes in the frontal and prefrontal cortices in ALS with varying levels of cognitive deficit, ranging from ALS, through ALS-Ci, to ALS-FTD (16).

Utilizing magnetically labeled blood plasma as an endogenous contrast media, arterial spin labeling (ASL) perfusion is a non-invasive, rapidly repeatable method for quantitatively measuring cerebral blood flow (CBF). While ASL-MRI provides information comparable to FDG-PET (17), it does not involve exposure to radioactive tracers. In addition, ASL-MRI can be acquired in conjunction with structural MRI, which will reduce the cost. However, this technique is an under-recognized tool in the investigation of ALS, with only one article finding that the disease severity was correlated with brain perfusion as measured by ASL-MRI (18). However, this study did not set up any control groups, and the implementation of ASL at that time resulted in incomplete brain coverage, both of which limited its replicability and correct interpretation. The greater availability of 3.0 T MRI and the significant developments in image acquisition and data analysis have dramatically improved the reliability of the methodology. The aim of our study was to identify the perfusion signature of ALS patients using ASL-MRI, and to testify whether the disease spectrum has distinct perfusion relevance that might reflect the different degrees of cognitive deficit, as observed in VBM and FDG-PET studies.

## Patients and Methods

### Patients

This study was approved by the Research Ethics Committee of Peking Union Medical College Hospital. Patients with ALS who agreed to undergo both neuropsychological assessment and MRI scanning were recruited after informed written consent had been obtained from themselves or their guardians. All patients fulfilled the revised El Escorial diagnostic criteria for clinically definite, probable, or lab-supported probable ALS (19, 20). ALS-FTD patients also fulfilled the diagnostic criteria for FTD (21). Familial ALS and patients with a history of neurological conditions affecting cognition (major stroke, traumatic head injuries, and severe active epilepsy), substance dependence, severe psychiatric illness, and use of high-dose psychoactive medications were not enrolled. A total of 20 age- and education-matched healthy controls (HCs) were also included in the study.

Demographic and clinical characteristics of included patients, including the age at the time of MRI, gender, site of onset, education level, and disease duration (defined as the time interval between onset and diagnosis) were collected. Disease severity was assessed by the revised ALS functional rating scale (22). All participants underwent a series of neuropsychological batteries. Selected tests included the following: category and phonemic verbal fluency, Stroop color–word interference effect, clock drawing test, paired associate word learning of the clinical memory test, episodic memory of the modified Wechsler memory scale, symbol digit modalities test, digit span of the Wechsler adult intelligence scale, repetition, and copy subsets of the aphasia battery of Chinese and the frontal assessment battery (23). The depression and anxiety evaluation was based on the Hamilton depression and anxiety rating scale. Details regarding neuropsychological battery testing and cognitive classification have been reported previously (23); see Data Sheet S1 in Supplementary Material. Patients were subdivided into the following three cognitive subgroups: (1) ALS with normal cognition (ALS-Cn), (2) ALS with cognitive deficits not fulfilling the criteria for FTD, but presenting with impairment on a minimum of two tests of executive or non-executive functions (ALS-Ci), and (3) ALS-FTD. Diagnosis of ALS with behavioral impairment (ALS-Bi) required the individual met at least two non-overlapping supportive diagnostic features from the Neary criteria (2). ALS in association with Alzheimer’s disease (AD), vascular dementia, or mixed dementia was considered to be ALS with non-FTD dementia (3). ALS with impairment on one executive and/or one non-executive test was defined as ALS with non-classifiable cognitive impairment (8). ALS with prevalent behavioral impairment but not meeting the criteria for FTD, ALS with non-classifiable cognitive impairment, or ALS with non-FTD dementia was not detected in this study.

### Image Acquisition

Images were acquired on a 3.0 T magnetic resonance system (Discovery MR 750, GE) with an 8-channel phase array head coil. A sagittal T1-weighted three-dimensional (3D) fast spoiled gradient echo sequence (TR = 8.2 ms, TE = 3.2 ms, flip angle = 12°, matrix = 256 × 256, FOV = 25.6 cm × 25.6 cm, slice thickness = 1.0 mm, no slice gap) was applied to acquire structural images of the whole brain. Images of 3D pseudocontinuous ASL (PCASL) were collected using the following protocol: 8 arms with 512 points in each spiral arm, bandwidth = ±62.5 kHz, slice thickness = 4 mm, postlabel delay = 2,025 ms, image reconstruction matrix = 128 × 128, FOV = 24 cm, and number of excitations = 2. Fast axial T1 FLAIR with the same section as ASL and other conventional sequences (T2WI, FLAIR, DWI, and T2*WI) were also performed to exclude major neurological disorders such as tumor, stroke, or advanced WM disease.

### Data Analysis

Imaging data were postprocessed using SPM12 implemented in Matlab 2015a. VBM was performed to observe GM changes using the DARTEL method, including the standard procedure of segmentation template creation based on all participants, normalization to Montreal Neurological Institute space, and smoothing with an 8 mm full width at half maximum (FWHM) filter, following the VBM tutorial.^1^ CBF maps were generated with the software provided by the scanner vendor, and the following processing procedures were performed including coregistration, first normalization, customized CBF template creation, second normalization, and smoothing (FWHM = 8 mm). Global voxel-based analyses of GM volume changes and CBF were performed using the statistical analysis module of the DPABI package^2^ (24), including analysis of covariance (ANCOVA) of the four groups and two-sample *t*-tests between paired groups, considering sex, age at MRI scan, and education years as text covariates. When we compared the CBF differences among the groups, GM maps were also considered as image covariates to control for the GM loss. All comparisons were tested for significance at *p* < 0.05 voxel-wise-corrected for multiple comparisons using false discovery rate (FDR). If significant clusters were not found, a more liberal voxel-wise threshold at *p* < 0.001 uncorrected with an extent threshold of 100 voxels was adopted.

### Statistical Analysis

Using the SPSS 22.0 statistical package, demographic and clinical data of patients in different groups were compared using the χ^2^ test or Fisher’s exact test for discrete variables and a two-sample *t*-test for continuous variables following a normal distribution. For variables that did not follow a normal distribution, Mann–Whitney *U* tests were carried out.

## Results

### Demographic and Clinical Data

A total of 55 patients with ALS were included: 27 patients were diagnosed with ALS-Cn, 17 with ALS-Ci (12 with executive dysfunction and 5 with non-executive dysfunction), and 11 with ALS-FTD. The demographic and clinical features of included patients are summarized in Table 1. We found no difference in sex, site of onset, mean age at the time of MRI, mean education years, median disease duration, disease severity, or progression rate between the subgroups. The results of the cognitive performance tests for each subgroup can be found in Data Sheet S1 in Supplementary Material.

**Table 1:** Demographic and clinical characteristics of included patients.

	HCs (*n* = 20)	ALS-Cn (*n* = 27)	ALS-Ci (*n* = 17)	ALS-FTD (*n* = 11)
Mean age at MRI (SD), years	55.3 (8.4)	52.5 (10.8)	54.0 (8.3)	60.0 (12.7)
Male/female	7/13	15/12	8/9	8/3
Education (SD), years	10.2 (3.4)	10.3 (3.4)	8.6 (2.6)	11.2 (4.8)
Onset, bulbar/limb	–	8/19	3/14	6/5
Median disease duration (IQR), month	–	12 (7–40)	14 (6–38)	11 (2–31)
Median ALSFRS-R total score (IQR)	–	42 (35–47)	41 (30–47)	42 (35–46)
Median progression rate (IQR)	–	0.40 (0.10–1.25)	0.50 (0.09–2.25)	0.72 (0.13–1.20)

*ALS-Cn, amyotrophic lateral sclerosis with normal cognition; ALS-Ci, ALS with cognitive impairment; ALSFRS-R, ALS functional rating scale—revised; FTD, frontotemporal dementia; IQR, interquartile range; HCs, healthy controls*.

*The disease progression rate was calculated according to the formula of (48 − ALSFRS-R score)/disease duration (month)*.

### Group Comparisons of GM Data

**Figure 1:**
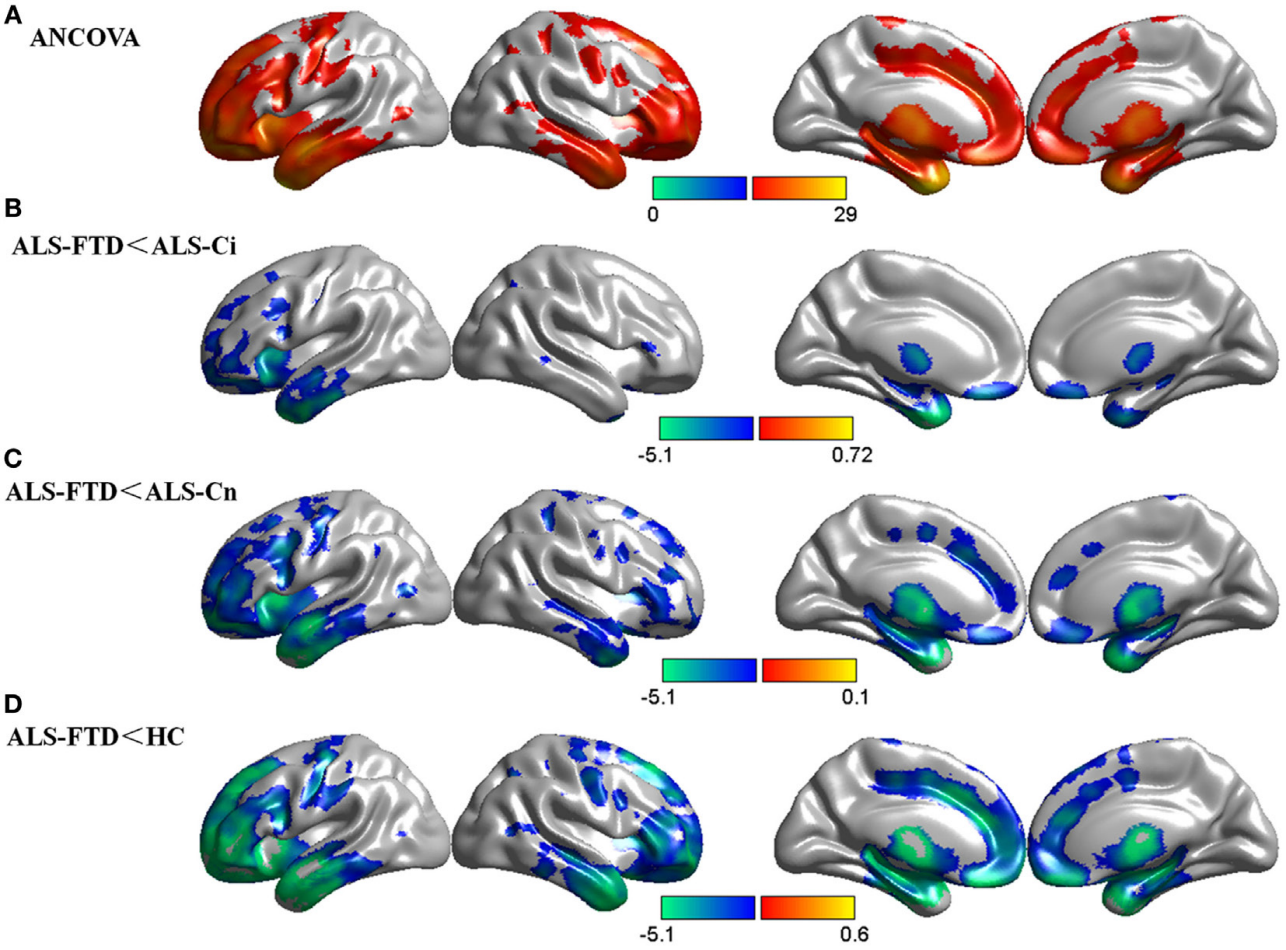
Group comparisons of gray matter (GM) data. **(A)** GM differences among the four subgroups. **(B)** GM reduction in amyotrophic lateral sclerosis (ALS)-frontotemporal dementia (FTD) patients compared with that in ALS with cognitive impairment (ALS-Ci) patients. **(C)** GM reduction in ALS-FTD patients compared with that in ALS with normal cognition (ALS-Cn) patients. **(D)** GM reduction in ALS-FTD patients compared with that in healthy controls (HCs). The color bar indicates the range of the *F* **(A)** or *t* **(B–D)** value. For all, *p* < 0.05 false discovery rate corrected. The results are visualized using Brant toolbox (version 2.0, http://brant.brainnetome.org/en/latest/).

The ANCOVA revealed a significant main effect of cognitive diagnosis, and regions of GM differences include the frontal lobe, temporal lobe, precentral gyrus, cingulate gyrus, and thalamus, all bilaterally (*p* < 0.05 FDR-corrected, Figure 1A). The differences primarily stemmed from the fact that patients with ALS-FTD exhibited greater atrophy in these areas than did the other three groups (*p* < 0.05 FDR-corrected, Figures 1B–D). It is noteworthy that a similar atrophy pattern was found in the comparisons of ALS-FTD vs. ALS-Ci, ALS-FTD vs. ALS-Cn, and ALS-FTD vs. HCs, with the size of the areas of involvement and the significance of the differences increasing in the above order. The whole-brain voxel-wise comparison between ALS-Cn vs. HC, ALS-Ci vs. HCs, and ALS-Ci vs. ALS-Cn revealed no differences at the set statistical threshold (*p* < 0.001 uncorrected). Please refer to Data Sheet S2 in Supplementary Material for details regarding anatomical regions and brain coordinates for each contrast.

### Group Comparisons of CBF Data

**Figure 2:**
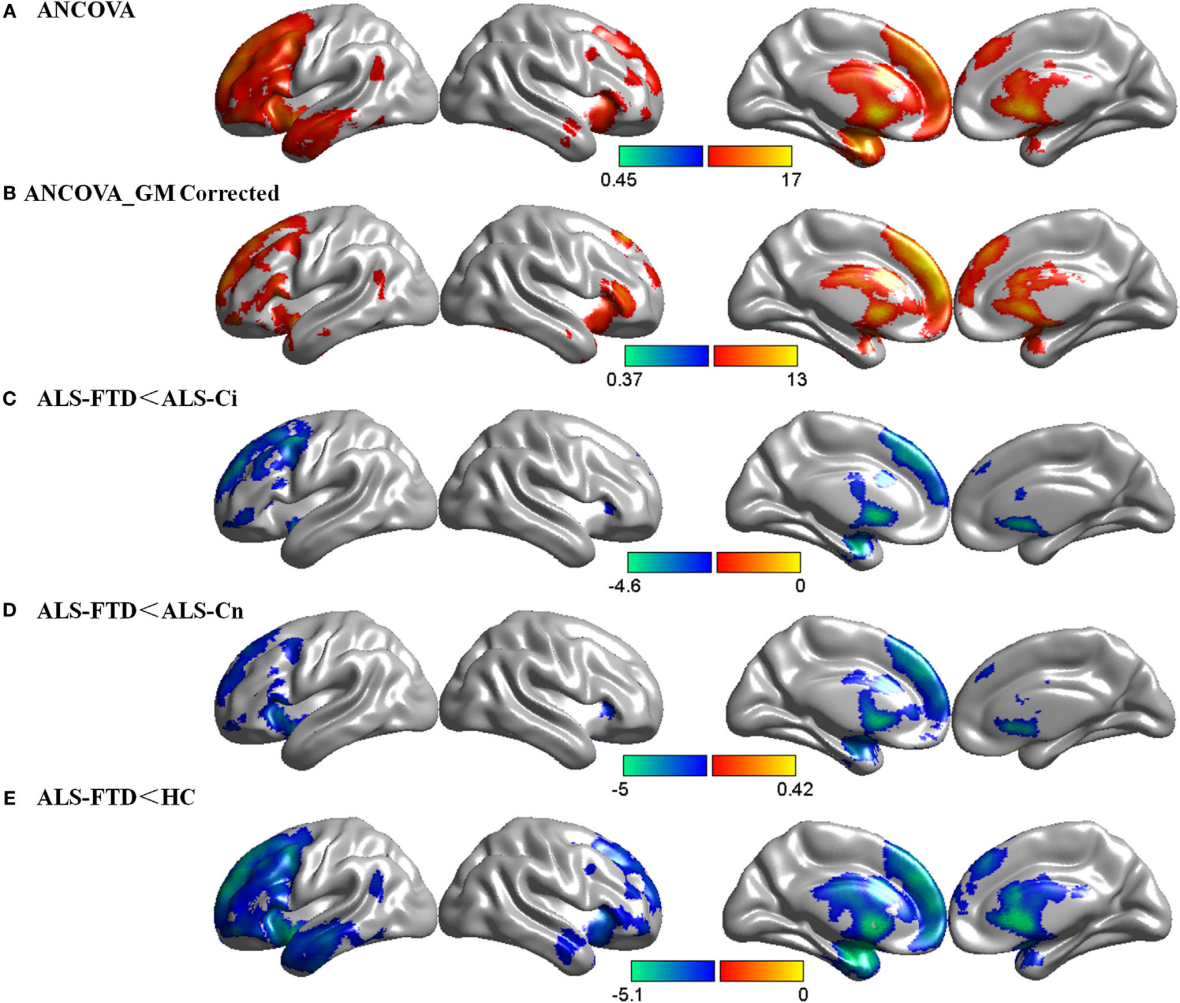
Group comparisons of cerebral blood flow (CBF) data. **(A)** CBF differences among the four subgroups without gray matter (GM) correction. **(B)** CBF differences among the four subgroups with GM correction. **(C)** CBF decrease in amyotrophic lateral sclerosis (ALS)-frontotemporal dementia (FTD) patients compared with that in ALS with cognitive impairment (ALS-Ci) patients. **(D)** CBF decrease in ALS-FTD patients compared with that in ALS with normal cognition (ALS-Cn) patients. **(E)** CBF decrease in ALS-FTD patients compared with that in healthy controls (HCs). The color bar indicates the range of the *F* **(A,B)** or *t* **(C–E)** value. For all, *p* < 0.05 false discovery rate-corrected. The results are visualized using Brant toolbox (version 2.0, http://brant.brainnetome.org/en/latest/).

There was a significant main effect of cognitive diagnosis in the ANCOVA, and regions of CBF differences include the bilateral frontal lobe, insular lobe, corpus callosum, and caudate, predominantly in the left hemisphere (*p* < 0.05 FDR-corrected, Figure 2A). Using GM loss as a covariate, the results of CBF were consistent with those without GM correction but with smaller areas of involvement (*p* < 0.05 FDR-corrected, Figure 2B). In addition, a similar pattern of hypoperfusion was found in the comparisons of ALS-FTD vs. ALS-Ci, ALS-FTD vs. ALS-Cn, and ALS-FTD vs. HCs, with the differences being most significant between ALS-FTD and HCs (*p* < 0.05 FDR-corrected, Figures 2C–E). Details regarding anatomical regions and brain coordinates for each contrast can be found in Data Sheet S2 in Supplementary Material.

**Figure 3:**
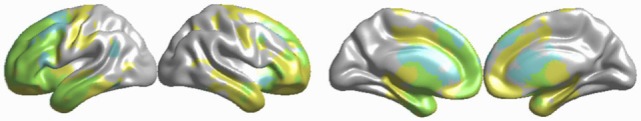
Concordance and discordance between brain atrophy and hypoperfusion in amyotrophic lateral sclerosis (ALS)-frontotemporal dementia (FTD) patients. Yellow indicates gray matter (GM) loss in ALS-FTD patients compared with that in healthy control (HC), ALS-Ci, and ALS-Cn; blue indicates cerebral blood flow (CBF) decrease in ALS-FTD patients compared with that in HC, ALS with cognitive impairment (ALS-Ci), and ALS with normal cognition (ALS-Cn); green indicates overlap between GM loss and CBF decrease.

Figure 3 illustrates that the majority of the areas with significant hypoperfusion also exhibited GM loss. However, sections of the right prefrontal and temporal lobes, bilateral precentral gyrus, cingulate gyrus, and bilateral thalamus exhibited only atrophy, and the bilateral caudate and sections of the medial prefrontal cortex exhibited only hypoperfusion. The number of voxels corresponding to GM loss accounted for 14.9% of the total number of GM voxels, and the number of voxels corresponding to decreased CBF accounted for 27.6% of the total number of GM voxels, while the proportion of overlapping regions was 8.2%. Hence, GM loss and CBF reduction were not independent, and the overlap was not due to chance alone.

## Discussion

To date, this is the first study using ASL to assess the voxel-based CBF of ALS patients with varying levels of cognitive deficit. Combined with the VBM study, we identified a similar pattern of frontal and temporal lobe hypoperfusion and GM loss among ALS-FTD patients compared with the patterns observed in HCs, ALS-Ci, and ALS-Cn patients, and the alterations were more significant in the left hemisphere. Perfusion abnormalities were detected in many of the same brain areas wherein structural alterations occurred, although isolated hypoperfusion and atrophy were also detected in some GM areas.

### VBM Study

A voxel-wise meta-analysis of VBM studies demonstrated that the entire cohort of ALS patients exhibited a significant GM volume reduction in the bilateral precentral gyrus and the frontal and temporal lobes (10). However, compared with HCs, no significant GM loss was observed in ALS-Ci and ALS-Cn patients in our study, which corroborates the results of previous studies (25–32). There are several possible explanations for the conflicting results. First, the pathological abnormalities of some ALS patient can be very subtle, thus reflecting the intrinsic heterogeneity of the pathological process. The different clinical characteristics, including disease duration and physical disability, particularly the upper motor neuron involvement of the patient cohorts, may have contributed to the divergence. Moreover, from a neuropathological perspective, reactive gliosis in the deep layers of the motor cortex might occur to a sufficient extent to “conceal” tissue loss (33), which is another possible explanation. Finally, the discrepancy could partially result from methodological factors (e.g., cortical thickness measurement vs. VBM) related to the MRI analysis.

When the comparisons were conducted within the ALS cognitive subgroups, we found no difference between ALS-Cn and ALS-Ci, while previous investigations have reported that ALS-Ci patients exhibited intermediate cortical atrophy and thinning between pure ALS and ALS-FTD (11, 12). However, the differences in GM changes between the ALS-FTD and ALS-Ci patients overlapped with those found in the comparisons of ALS-FTD vs. ALS-Cn and ALS-FTD vs. HC, and the changes became more widespread in this order. Therefore, the more severe the cognitive decline, the larger the size of the cluster and the more significant the group differences. In other words, there was a trend toward GM loss in ALS-Ci compared with ALS-Cn, although no significant cluster was observed at the set statistical threshold. It should also be noted that, unlike the VBM findings, the ALS-Ci and ALS-Cn groups were almost identical in their comparison with the ALS-FTD group in the CBF analyses. There are three possible reasons for such controversial results. First, the sample size in our study was relatively small, particularly in the ALS-Ci group. Nevertheless, under a relatively stringent cutoff of neuropsychological assessments (2SD below HCs), the rate of cognitive impairment and comorbidity of FTD in ALS in our cohort (16 and 4.7%, respectively) (23) appeared to be markedly lower than that in Europeans and Americans (4–8). The spectrum of cognitive impairment in Korea also supports a relatively lower prevalence of cognitive impairment in Asian patients than in Caucasian populations (34). Differences in genetic backgrounds and an early onset age in Asian patients might explain this prevalence disparity (35, 36). Second, the level of cognitive impairment varied greatly in the ALS-Ci group, ranging from barely perceptible disorders immediately below average function to dysfunction that is sufficiently apparent for the patients or their caregivers to notice in their daily activities. Such heterogeneity would lessen the discrepancies between ALS-Ci and ALS-Cn and might produce inconsistent findings in research on imaging measures pertaining to cognitive impairment in ALS. Third, WM degeneration in ALS patients might be the initial change leading to cognitive impairment. This notion is supported by evidence suggesting that reduction of WM integrity in the frontal, temporal, and parietal long-range tracts is highly associated with neuropsychological symptoms in patients with ALS, and DTI measures of WM involvement are the most sensitive markers of extramotor impairments (14, 15).

In keeping with MRI studies of patients with ALS-FTD, a distributed GM volume reduction across the whole brain was observed in the ALS-FTD patients in our study, regardless of the comparative group. In addition, in all brain regions, there was a trend toward a more severe GM loss compared with, in increasing order, ALS-Ci patients, ALS-Cn patients, and HCs, particularly in the left frontal and prefrontal lobes and the bilateral temporal lobe. Degeneration of the frontal and prefrontal cortices is associated with executive dysfunction and behavioral disorders. The temporal lobes subserve various cognitive domains, including processing of color information and word and face recognition. Atrophy of the limbic system might lead to emotional dysregulation and memory deficits. All of the symptoms mentioned earlier were noted in the majority of the ALS-FTD patients in this study. We also detected significant subcortical GM loss within the basal ganglia, which are directly connected to the key cortical regions involved in ALS pathology. This finding is in agreement with a previous MRI study that highlighted widespread basal ganglia involvement along the ALS/FTD spectrum and suggested that the degree of subcortical GM loss in ALS is closely associated with cognition (13). The cerebellum was not considered to be significantly involved in ALS, but recent studies have yielded controversial results with regard to these areas. While many previous studies (10–12, 37) and this study found no differences within the ALS cognitive subgroups, one study reported specific patterns of cerebellum atrophy in ALS, ALS-FTD, and FTD patients (38), which need to be further investigated.

### ASL Study

Since CBF is closely related to brain metabolism, ASL-MRI might depict functional deficiencies in a manner similar to FDG-PET. Similar patterns of hypoperfusion and hypometabolism in regions typically associated with AD and FTD were identified using this technique, indicating that ASL-MRI is in good accordance with FDG-PET (39). However, this technique has not been widely applied in the investigation of ALS. For the first time, we utilized 3D PCASL to explore the perfusion signature of the cognitive spectrum of ALS. In a study that assessed brain perfusion as a possible marker of disease severity in ALS, the investigators adopted pulsed ASL (PASL) sequences on a 1.5 T MR system (18). Continuous ASL (CASL) continuously labels blood plasma as it passes through a labeling plane, while PASL uses short radiofrequency pulses to label blood and tissue selectively. PCASL integrates these approaches in a manner wherein many short pulses simulate the continuous labeling of CASL, which greatly reduces cardiac noise and increases retest reliability. This reproducibility advantage of PCASL occurs at the whole-brain GM level, and more recent advances with 3D sequences allow for background suppression of static tissue water, thereby improving the sensitivity of PCASL to CBF (17).

We also examined whether the CBF results could be influenced by regional GM changes. FTD is known to be associated with widespread GM loss in many brain regions, primarily the frontal and temporal regions. GM loss may lead to an artificial reduction in measured blood perfusion. When comparing CBF differences among the cognitive subgroups and controls, this issue could potentially be crucial due to group differences in the degree of regional atrophy. In this study, after GM correction, we found that the CBF results were slightly altered, primarily in areas of involvement in the left frontal and prefrontal lobe that were smaller, but the patterns were approximately consistent with those before correction. This implies that our CBF results could reflect the intrinsic brain perfusion alterations in these patients.

### Discrepancy Between GM and CBF Changes

In our study, regions with reduced CBF detected in patients with ALS-FTD in extramotor regions, particularly the left prefrontal and temporal cortices, correlated with the structural changes observed in VBM studies. Interestingly, these regions are considered to be particularly vulnerable in patients with FTD. Our findings are supported by the results of a study comparing structural brain MRI and metabolic FDG-PET changes in the cortical regions of patients with ALS-FTD, in which significant reductions in cerebral glucose metabolism rates and structural changes in brain regions were concomitantly observed (37). It should be noted that this global concordance between perfusion/metabolism and structural changes observed in ALS-FTD patients disagrees with findings in AD patients. Several studies have explored the perfusion signature in early and prodromal AD using ASL-MRI or SPECT and have reported that regions of significant hypoperfusion disaccorded with regions of significant GM loss (40–42). Such discordances have repeatedly been reported in FDG-PET studies and were attributed to the possibility that the decreased CBF in the posterior cingulate cortex reflects remote effects caused by neuronal degeneration in the medial temporal structures (42–44). Contrasts of CBF changes in AD and ALS/FTD also exhibited disease-specific perfusion patterns, in which decreased CBF was observed in the posterior cingulate cortex and precuneus in AD, in the frontal gyrus and insula in FTD, and in the frontal cortex in ALS (45). The above evidence is suggestive of distinct pathophysiological procedures in AD and ALS/FTD along the neurodegenerative course.

However, isolated GM loss and CBF decrease were also detected in ALS-FTD patients in our study. Such local discordance between brain perfusion/metabolism and structural changes was observed in patients with ALS and the behavioral variant FTD as well (37, 46–48) (Table 2). The finding of discordant brain atrophy in the absence of hypoperfusion can be interpreted as a result of brain tissue loss outweighing neuronal loss, compensatory mechanisms being at work, and surviving neurons functioning normally to maintain normal levels of perfusion/metabolism (46, 47). Pathological studies have revealed that astrocytosis and/or microglial activation occurs in the early stages of ALS and FTD before frank neuron loss, which supports this theory (49, 50). We also found greater hypoperfusion than GM atrophy in the bilateral caudate and sections of the medial prefrontal cortex. This finding points to a genuine functional change over neuronal loss and might also be relevant to the remote effects mentioned earlier in AD patients. The remote effect of GM atrophy on brain metabolism, namely, diaschisis, refers to metabolic activity alterations in neurons that are anatomically or functionally connected to structural abnormalities (47). This situation will not continue for long, as the degeneration of disconnected neurons is in progress, giving rise to different patterns of brain structural and metabolic impairment, depending on the disease stage (47). A longitudinal study also reported CBF alterations in presymptomatic FTD patients (individuals with MAPT or GRN mutations) independent of GM atrophy, in which individuals with the greatest decrease developed symptoms during the follow-up, thus indicating that perfusion deficits may exist at the presymptomatic stage of the disease before substantial atrophy is present (51). Thus, ASL can provide early information pertaining to the lesion burden in patients on the ALS/FTD spectrum, even at the presymptomatic stage.

**Table 2:** Summary of studies comparing perfusion/metabolism and structural changes in patients on the ALS/FTD spectrum.

Reference	Subjects (sample size)	Technique	Isolated atrophy	Isolated perfusion/metabolism abnormalities
This study	ALS-FTD (11)	VBM and ASL-MRI	Right prefrontal and temporal lobes, bilateral precentral gyrus, cingulate gyrus, and bilateral thalamus	Bilateral caudate

Rajagopalan and Pioro (37)	ALS-FTD (18)	VBM and FDG-PET	Left insula	Left postcentral gyrus

Buhour et al. (46)	ALS (37)	VBM and FDG-PET	Bilateral temporal pole, left hippocampus, parahippocampus, inferior temporal gyrus, and calcarine	Left superior medial frontal cortex

Buhour et al. (47)	bvFTD (15)	VBM and FDG-PET	Right putamen and amygdala, left insula, and superior temporal gyrus	Left caudate and anterior cingulate cortex

Shimizu et al. (48)	bvFTD (28)	VBM and ASL-MRI	Bilateral premotor cortex	None

*ALS, amyotrophic lateral sclerosis; bvFTD, behavioral variant of frontotemporal dementia; FDG-PET, 18F-2-fluoro-2-deoxy-d-glucose-PET; VBM, voxel-based morphometry; ASL, arterial spin labeling; FTD, frontotemporal dementia*.

### Limitations

Despite the promising results, the limitations of this study should be considered. First, the main limitation is the relatively small numbers of patients with ALS-Ci and ALS-FTD. It would be beneficial to replicate these findings in larger independent samples and to establish the clinical value of ASL in managing ALS or FTD. Second, although we did not select the tests requiring hand function for a great number of patients who were affected by motor disability, and verbal tests were selected instead, we did not adjust for patients with bulbar dysfunction, whose scores might be lowered consequently. In addition, to avoid patient fatigue, we did not include the assessment of premorbid IQ, which is an important predictor of cognitive reserve, despite its minor impact on executive function. Third, as this is a cross-sectional study, patients were studied at a single time point only, and brain alterations of ALS cognitive subgroups over time remain largely unexplored. Nevertheless, ALS patients with cognitive deficits, including ALS-FTD patients, have shorter lifespans than patients without cognitive or behavioral impairment (8, 34), which poses a challenge for long-term follow-ups. Fourth, we detected no significant difference in both GM and CBF data among the ALS-Ci, ALS-Cn, and HC groups, while the gradient of brain damage observed was based on the visual inspection of their contrasts with the ALS-FTD group. Finally, when the two imaging methods were compared, we performed the visual analysis by superimposing the corresponding maps and conducting preliminary statistics without more rigorous comparisons, which limited the interpretation of the results.

### Conclusion

In conclusion, the cognitive status of ALS patients is associated with different patterns of GM atrophy and cerebral perfusion, lending support to the hierarchical brain changes observed in patients on the ALS/FTD spectrum. Our findings also highlight the complex relationships and regional heterogeneity between GM atrophy and hypoperfusion in ALS-FTD patients. While the majority of the perfusion alterations largely overlapped with the structural changes, greater GM atrophy than hypoperfusion might suggest that the brain tissue loss did not involve metabolically active cells or that the perfusion of the remaining cells was higher than expected. On the other hand, greater hypoperfusion than GM atrophy could reflect either a diaschisis mechanism or an early stage of metabolic neuronal failure before cell death. Finally, our study emphasizes that any single imaging technique alone does not allow a complete understanding of the brain alterations in ALS. ASL-MRI can identify early regional spreading of pathological changes and is beneficial for exploring the pathophysiological basis of cognitive impairment in ALS along the disease course.

## Ethics Statement

This study was carried out in accordance with the recommendations of Good Clinical Practice and applicable local regulations with written informed consent from all subjects. All subjects gave written informed consent in accordance with the Declaration of Helsinki. The protocol was approved by the Research Ethics Committee of Peking Union Medical College Hospital.

## Author Contributions

Study concept and design: LC and FF. Acquisition of data: DS, BH, YX, BC, PP, XL, HT, HF, KZ, SL, JG, and ML. Analysis and interpretation of the data; drafting of the manuscript: DS and BH. Study supervision: JG, ML, FF, and LC. Critical revision of the manuscript: LC.

## Conflict of Interest Statement

The authors declare that the research was conducted in the absence of any commercial or financial relationships that could be construed as a potential conflict of interest.

## Footnotes

^1^http://www.fil.ion.ucl.ac.uk/~john/misc/VBMclass10.pdf (Accessed: April 13, 2017).

^2^http://rfmri.org/dpabi (Accessed: April 13, 2017).
