# Supplementary material for: Brain Structural and Perfusion Signature of Amyotrophic Lateral Sclerosis With Varying Levels of Cognitive Deficit
Source: Front Neurol. 2018 May 24;9:364. doi: 10.3389/fneur.2018.00364 (PMC5976730; doi:10.3389/fneur.2018.00364)
Supplement: Supplementary file 1 [file data_sheet_1.DOCX]

Table 1 Cognitive domains and corresponding neuropsychological tasks

| Cognitive domains | neuropsychological tasks |
| --- | --- |
| Executive function | Phonemic verbal fluency |
|  | Category verbal fluency |
|  | Backward digit span of the Wechsler Adult Intelligence Scale |
|  | the Stroop Color Word Task |
|  | the Clock Drawing Test |
| Attention | Forward digit span of the Wechsler Adult Intelligence Scale |
|  | the Symbol Digit Modalities Test |
| Memory | Paired associate word learning of the Clinical Memory Test |
|  | Episodic memory of the modified Wechsler Memory Scale |
| Language | Repetition subset of the Aphasia Battery of Chinese |
|  | Category verbal fluency |
| Visuospatial function | Copy subset of the Aphasia Battery of Chinese |
|  | the Clock Drawing Test |
| Depression | Hamilton depression rating scale |
| Anxiety | Hamilton anxiety rating scale |

Two standard deviations (SD) below the mean of HC was set as the cutoff value for each neuropsychological test except for the Clock Drawing Test (4 score method was adopted and 3 was set as the cut-off). At least 2 tests in one domain scored below the cutoff reached the threshold of impairment (cognitive domains and corresponding tests are shown in Table 1). Non-demented subjects who displayed impairment in executive function would be identified as ALS with executive cognitive impairment (ALS-ECi). And those with impairment in any non-executive domain but without executive dysfunction would be defined as ALS with non-executive cognitive impairment (ALS-NECi). Both ALS-ECI and ALS-NECI were regarded as ALS with cognitive impairment (ALS-Ci). The remaining patients were regarded as ALS with normal cognition (ALS-Cn).

Since the neuropsychological data of ALS-FTD were incomplete, we only compared the neuropsychological performances within HC, ALS-Cn and ALS-Ci. One-way ANOVA (normal distribution) or Kruskal-Wallis (abnormal distribution) tests were adopted.

Table 2 Neuropsychological performances of included patients

| neuropsychological tasks | HC  (n=20) | ALS-Cn  (n=27) | ALS-Ci  (n=17) | ALS-FTD  (n=11) | P Value |
| --- | --- | --- | --- | --- | --- |
| Phonemic verbal fluency | 5.8±2.6 | 5.2±2.2 | 5.2±2.7 | 2.3±0.8* | 0.864 |
| Category verbal fluency | 18.0±4.3 | 17.3±3.8 | 13.9±3.0 | 6.8±0.9* | 0.024 |
| Backward digital span of the WAIS | 5.5±1.2 | 5.1±1.2 | 3.6±1.4 | 3.2±0.9† | 0.032 |
| Stroop interference effect | 0.80±0.2 | 0.75±0.4 | 0.77±0.2 | NA | 0.249 |
| Episodic memory of modified WMS | 6.7±1.3 | 7.0±1.5 | 4.7±2.2 | NA | 0.015 |
| Paired associate word learning of the CMT | 11.0±4.2 | 10.5±3.4 | 7.7±2.2 | NA | 0.032 |
| Forward digital span of the WAIS | 8.0±0.9 | 8.2±1.1 | 7.3±1.2 | 6.0±1.4† | 0.053 |
| the Symbol Digit Modalities Test | 45.7±10.8 | 44.5±10.0 | 31.3±9.1 | 16.8±4.9† | 0.011 |
| Errors in repetition of the ABC | 2 (0-5) | 1 (0-4) | 2 (1-5) | NA | 0.787 |
| Copy of the ABC | 9.2±1.0 | 9.0±2.5 | 7.9±2.4 | 5.0±2.2† | 0.433 |
| Hamilton depression rating scale | 1 (0-2) | 3 (1-5) | 3 (0-4) | NA | 0.158 |
| Hamilton anxiety rating scale | 2 (0-3) | 1 (0-4) | 1 (0-4) | NA | 0.877 |

* Nine persons completed this test.

† Eight persons completed this test.

NA: less than five persons completed this test.

ABC, Aphasia Battery Chinese; ALS, amyotrophic lateral sclerosis; CMT, Clinical Memory Test; HC, healthy controls; WAIS, Wechsler Adult Intelligence Scale; WMS, Wechsler Memory Scale; stroop interference effect was calculated according to the formula of (Stroop C time/Stroop correct number-Stroop B time/Stroop correct number).

Data were means ± SD or median (IQR)
