# Supplementary material for: Brain Structural and Perfusion Signature of Amyotrophic Lateral Sclerosis With Varying Levels of Cognitive Deficit
Source: Front Neurol. 2018 May 24;9:364. doi: 10.3389/fneur.2018.00364 (PMC5976730; doi:10.3389/fneur.2018.00364)
Supplement: Supplementary file 2 [file data_sheet_2.DOCX]

Table1. Group comparisons of GM data

|  | Cluster size | Peak in the cluster (MNI) | | | Regions Encompassed (AAL) | F- or T -value |
| --- | --- | --- | --- | --- | --- | --- |
|  |  | X | Y | Z |  |  |
| ANCOVA | 115193 | -29 | -5 | -42 | Temporal_Inf_L | 28.92 |
|  |  |  |  |  | Frontal_Mid_L |  |
|  |  |  |  |  | Temporal_Mid_L |  |
|  |  |  |  |  | Frontal_Sup_R |  |
|  |  |  |  |  | Frontal_Inf_Orb_L |  |
|  |  |  |  |  | Frontal_Sup_L |  |
|  |  |  |  |  | Frontal_Inf_Tri_L |  |
|  |  |  |  |  | Insula_L |  |
|  |  |  |  |  | Precentral_L |  |
|  |  |  |  |  | Putamen_L |  |
|  | 945 | 48 | -9 | 45 | Precentral_R | 8.57 |
|  | 255 | 41 | 8 | 27 | Frontal_Inf_Oper_R | 8.03 |
| ALS-FTD<ALS-Ci | 23669 | -30 | 9 | -35 | Temporal_Pole_Mid_L | -5.04 |
|  |  |  |  |  | Temporal_Inf_L |  |
|  |  |  |  |  | Insula_L |  |
|  |  |  |  |  | Temporal_Mid_L |  |
|  |  |  |  |  | Frontal_Inf_Orb_L |  |
|  |  |  |  |  | Temporal_Pole_Mid_L |  |
|  |  |  |  |  | Fusiform_L |  |
|  | 1430 | -9 | -8 | 3 | Thalamus_L | -4.61 |
|  |  |  |  |  | Thalamus_R |  |
|  | 561 | -38 | 14 | 23 | Frontal_Inf_Tri_L | -4.17 |
|  | 505 | 39 | -27 | -9 | Hippocampus_R | -4.19 |
|  | 325 | -24 | 38 | 38 | Frontal_Sup_L | -3.74 |
|  | 332 | -51 | -12 | 41 | Postcentral_L | -3.85 |
| ALS-FTD<ALS-Cn | 60824 | -37 | 11 | -25 | Temporal_Pole_Sup_L | -5.06 |
|  |  |  |  |  | Temporal_Mid_L |  |
|  |  |  |  |  | Insula_L |  |
|  |  |  |  |  | Frontal_Mid_L |  |
|  |  |  |  |  | Temporal_Inf_L |  |
|  |  |  |  |  | Putamen_L |  |
|  |  |  |  |  | Frontal_Inf_Orb_L |  |
|  | 1842 | -53 | -12 | 44 | Postcentral_L | -4.63 |
|  |  |  |  |  | Precentral_L |  |
|  | 438 | -17 | -8 | 57 | Frontal_Sup_L | -4.03 |
|  | 366 | 14 | -29 | 71 | Precentral_R | -3.80 |
|  | 294 | 36 | -38 | 53 | Parietal_Inf_R | -4.03 |
|  | 287 | 38 | 38 | 20 | Frontal_Mid_R | -4.13 |
|  | 256 | -5 | -2 | 47 | Cingulum_Mid_L | -4.27 |
|  | 242 | 9 | 39 | 20 | Cingulum_Ant_R | -3.91 |
| ALS-FTD<HC | 96045 | -45 | 12 | -28 | Temporal_Pole_Mid_L | -5.09 |
|  |  |  |  |  | Frontal_Mid_L |  |
|  |  |  |  |  | Temporal_Mid_L |  |
|  |  |  |  |  | Frontal_Sup_L |  |
|  |  |  |  |  | Frontal_Sup_R |  |
|  |  |  |  |  | Frontal_Inf_Tri_L |  |
|  |  |  |  |  | Temporal_Inf_L |  |
|  | 1246 | 45 | -12 | 42 | Precentral_R | -4.22 |
|  |  |  |  |  | Postcentral_R |  |
|  | 888 | 36 | -17 | 60 | Precentral_R | -4.05 |
|  | 796 | 11 | -5 | 45 | Cingulum_Mid_R | -3.94 |
|  | 256 | 47 | -60 | 9 | Temporal_Mid_R | -3.92 |
|  | 226 | 41 | 8 | 26 | Frontal_Inf_Oper_R | -3.93 |

All P<0.05, FDR corrected.

Table2. Group comparisons of CBF data

|  | Cluster size | MNI-space | | | Regions Encompassed | F- or T -value |
| --- | --- | --- | --- | --- | --- | --- |
|  |  | X | Y | Z |  |  |
| ANCOVA | 22437 | -42 | 18 | 4 | Insula_L | 18.35 |
|  |  |  |  |  | Frontal_Sup_Medial_L |  |
|  |  |  |  |  | Frontal_Mid_L |  |
|  |  |  |  |  | Frontal_Sup_L |  |
|  |  |  |  |  | Frontal_Sup_Medial_R |  |
|  | 545 | 47 | 32 | 12 | Frontal_Inf_Tri_R | 8.38 |
| ALS-FTD<ALS-Ci | 9811 | -8 | 12 | 0 | Caudate_L | -4.58 |
|  |  |  |  |  | Frontal_Mid_L |  |
|  |  |  |  |  | Frontal_Sup_Medial_L |  |
|  |  |  |  |  | Frontal_Sup_L |  |
|  |  |  |  |  | Precentral_L |  |
|  | 615 | -42 | 6 | -8 | Insula_L | -4.49 |
|  | 290 | 44 | 14 | -4 | Insula_R | -4.00 |
|  | 129 | 32 | 52 | 30 | Frontal_Mid_R | -3.91 |
| ALS-FTD<ALS-Cn | 11486 | -5 | 40 | 33 | Frontal_Sup_Medial_L | -4.93 |
|  |  |  |  |  | Frontal_Sup_Medial_L |  |
|  |  |  |  |  | Frontal_Sup_L |  |
|  |  |  |  |  | Caudate_L |  |
|  | 416 | -34 | 2 | 46 | Precentral_L | -3.84 |
|  | 365 | 52 | 21 | -32 | Temporal_Pole_Mid_R | -4.82 |
|  | 348 | 16 | -2 | -20 | ParaHippocampal_R | -4.20 |
|  | 285 | 42 | 18 | 0 | Insula_R | -3.82 |
| ALS-FTD<HC | 37020 | -44 | 6 | -3 | Insula_L | -5.04 |
|  |  |  |  |  | Frontal_Mid_L |  |
|  |  |  |  |  | Frontal_Sup_Medial_L |  |
|  |  |  |  |  | Frontal_Sup_L |  |
|  |  |  |  |  | Temporal_Mid_L |  |
|  |  |  |  |  | Frontal_Inf_Tri_L |  |
|  |  |  |  |  | Frontal_Mid_R |  |
|  | 239 | 54 | -6 | -14 | Temporal_Mid_R | -3.61 |
|  | 226 | 50 | 6 | 58 | Frontal_Mid_R | -4.22 |

All P<0.05, FDR corrected.
